# Single-Layered Microfluidic Network-Based Combinatorial Dilution for Standard Simplex Lattice Design

**DOI:** 10.3390/mi9100489

**Published:** 2018-09-25

**Authors:** Kangsun Lee, Choong Kim, Kwang W. Oh

**Affiliations:** 1 SMALL (Sensors and MicroActuators Learning Lab), Department of Electrical Engineering, The State University of New York at Buffalo, Buffalo, NY 14260, USA; kwangoh@buffalo.edu; 2 Sensor Solution Laboratory, LG Electronics, LG Science Park, Seoul 07796, Korea; 3 School of Mechanical and Automotive Engineering, Kyungil University, Daegu 712-701, Korea; ck.pistos@gmail.com; 4 Department of Biomedical Engineering, The State University of New York at Buffalo, Buffalo, NY 14260, USA

**Keywords:** combinatorial dilution, 3D simplex lattice design, microfluidic network, design of experiment (DOE), microfluidic spotting system

## Abstract

In this paper, we presented a straightforward strategy to generate 15 combinations of three samples based on an experimental simplex lattice design using a single-layer microfluidic network. First, we investigated the performances of the plain structural and the groove structural combinatorial devices by computational simulation (CFD-ACE+). The simulated output concentrations were extremely close to the desirable values within an absolute error of less than 1%. Based on the simulated designs, polydimethylsiloxane (PDMS) devices were fabricated with soft lithography and tested with fluorescent dye (sodium salt). The mixing results for 15 combinations showed good performance, with an absolute error of less than 4%. We also investigated two liquid handling methods (bottom–up and top–down) for high-throughput screening and assay. The liquid-handling methods were successfully accomplished by adding the systematic structured groove sets on the mixing channels.

## 1. Introduction

One-factor-at-a-time experiments are inefficient when compared with experiments in which factors are changed simultaneously and systematically. The use of a so-called design of experiments (DOE) can provide a great deal of information about all factors, including components and/or processes and their interactions [1,2]. The types of designed experiments commonly used in the DOE methodology are factorial designs, response-surface designs, mixture designs, and Taguchi robust designs [3]. The DOE methodology has recently been adopted by scientists and engineers who work in such domains as medical devices, pharmaceuticals, foods, materials science, and semiconductors [4,5,6,7,8]. They frequently deal with factorial DOE to screen the effect of each component, or mixture DOE to optimize mixtures for desired outcomes. Sophisticated robotics [9,10,11,12] or even manual pipetting is often used in the preparation of combinatorial mixtures with the relative concentrations of multiple-input solutions [13,14].

Microfluidic techniques have offered better alternatives in the generation of such combinatorial mixtures or dilutions in a relatively rapid, compact, low-cost, and high-throughput manner [15,16,17,18,19,20,21,22,23,24,25]. Microfluidic array formats are often considered [15,16,17,18], requiring the integration of vast arrays of multilayer polydimethylsiloxane (PDMS) pneumatic valves developed by Quake’s group [26,27] and treelike microfluidic concentration gradient generators introduced by Whiteside’s group [28]. For example, Ali’s group demonstrated a microfluidic combinatorial dilution device incorporating a 10 × 10 array of deep wells and two gradient generators capable of 100 discrete mixtures of three input solutions [15]. Other groups have used pneumatically isolated chambers for combinatorial mixing and screening of assays to produce various microfluidic arrays [16,17,18]. However, the incorporation of sophisticated pneumatic microfluidic design and fabrication technology is mandatory in order to produce a large number of combinatorial mixtures or dilutions. This often requires significant expertise that is not part of life-science training [14].

Another effort for the generation of combinatorial mixtures or dilutions has focused on continuously flowing microfluidic networks. As one of the early works in the field, Kitamori’s group nicely demonstrated four binary combinatorial mixtures with four reagents in a multilayer glass device [19]. Folch’s group also dramatically demonstrated combinatorial perfusion devices with a multiplex to control a large number of samples [20,21]. Tai’s group also demonstrated 3D microfluidic networks fabricated by a monolithic method using Parylene C for seven binary combinations with three samples [22]. Yang et al. showed a radial combinatorial dilution device to create a large number of dilution results [23]. In our previous work, we designed multilayer microfluidic combinatorial dilution devices that were able to generate seven combinations of three samples with various concentrations [24,25] using a simple electric-circuit analogy [29,30]. Although the use of continuous-flow microfluidic networks is appropriate for generating simple binary-based combinatorial mixtures because it has the prerequisite of much complicated multilayer design and fabrication technology, it has limited ability to produce complex combinatorial dilutions with multiple samples at multiple concentrations.

In addition to platforms using pneumatically isolated chambers or continuously flowing microfluidic networks, electrowetting-based digital microfluidic platforms could be well-suited for the creation of on-demand combinatorial mixtures or dilutions [31]. Recently, Wheeler’s group implemented a full factorial DOE [32] and a mixture DOE [33] using discrete droplets of liquid on the surface of an insulated two-dimensional array of electrodes. Although such a digital microfluidic format enables better droplet manipulation for combinatorial chemistry, it still requires an advanced integration of electrowetting-based microfluidics and electronics.

While a variety of new microfluidic devices have been developed for the generation of combinatorial mixtures or dilutions with multiple ingredients, a few older microfluidic platforms yet exist whose configurations are suitable for the optimization of mixtures via the DOE methodology. Experimental designs for such combinatorial mixture experiments are fundamentally different from those for binary-based factorial experiments. In a general mixture problem, it is assumed that the measured response depends only on the proportions of the ingredients in the mixture, not the amount of the mixture. Keeping this in mind, one important feature is an ability to systematically generate a large number of combinatorial mixtures with multiple samples (e.g., three ingredients) at multiple concentrations (e.g., proportions summing to one). For example, consider three samples (A, B, and C) in an *x*-*y*-*z* Cartesian coordinate system. The plane on which the proportions sum to one is a triangle-shaped slice in the form of a ternary plot; a corresponding plane equation of such a ternary plot is A + B + C = 1, where 0 ≤ A, B, C ≤ 1. When mixture ingredients are subject to the constraint that they must sum to one, the most common configurations are simplex designs in the DOE methodology.

In this paper, we have demonstrated a very simple continuous-flow microfluidic device that can generate 15 combinatorial mixture outputs with the relative concentrations of three-sample inputs (A, B, and C), representing a combinatorial mixture DOE configuration of a simplex design. In order to avoid any complex multi-layer configuration, a straightforward, single-layer, circular-type configuration has been used, thereby avoiding a highly sophisticated fabrication technique. This approach could also easily be extended to a larger number of combinatorial dilutions. In particular, a simplex design was used in order to generate 15-point, three-sample, combinatorial mixtures for which the number of equally spaced levels for each sample is five. We also conceptually showed how to deal with liquid-handling methods, including bottom–up and top–down, for a potential application of on-chip or off-chip combinatorial mixing and screening of high-throughput assays.

## 2. Concept and Design

**Figure 1:**
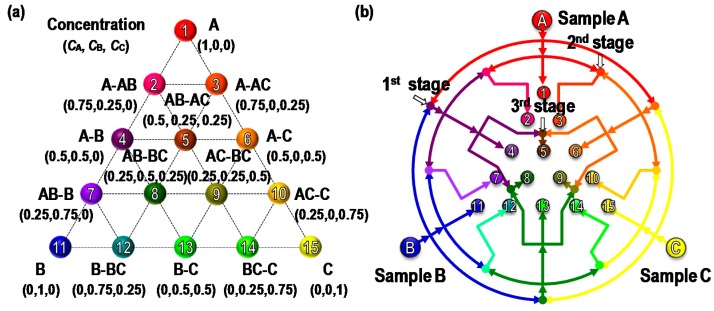
(**a**) The schematic of standard mixture design of experiment (DOE): Simplex lattice design configured by 15 mixture points for three samples (A, B, and C). (**b**) Two-dimensional microfluidic network for the proposed combinatorial dilutions of 15 mixtures by three samples.

**Figure 2:**
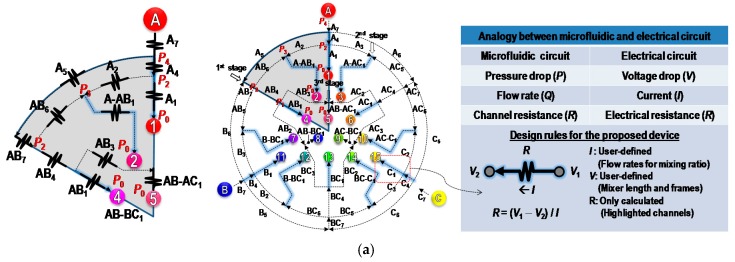

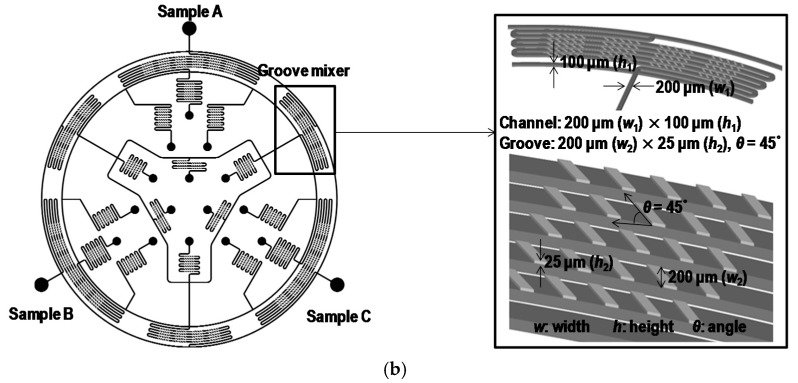
(**a**) Equivalent electrical circuit for modeling of proposed microfluidic network using electric-fluidic analogy. (**b**) Geometrical details of the proposed microfluidic device designed by the modeling.

**Figure 3:**
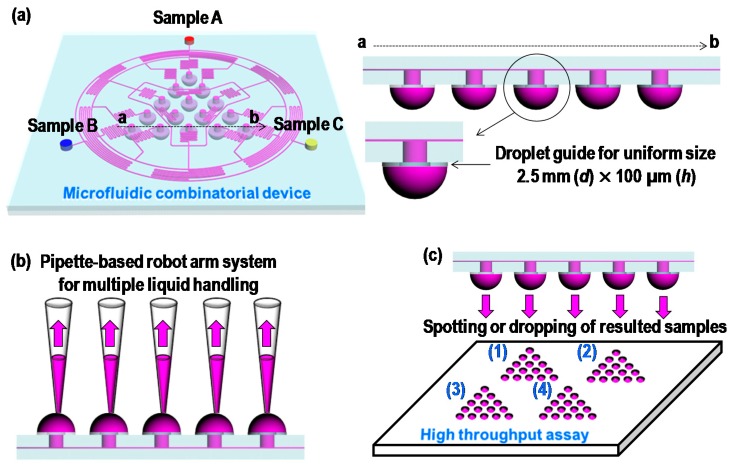
Potential liquid-handling methods using proposed microfluidic combinatorial device. (**a**) Cross-sectional view of proposed device for detailed design parameter for liquid handling. (**b**) Bottom–up method and (**c**) top–down method.

Figure 1a shows the combinatorial configuration of a standard simplex-lattice design for three input samples. The figure shows that the systematic arrangement of 15 combinatorial mixture points with three samples can effectively estimate the optimal mixture concentrations with minimal efforts. For the microfluidic combinatorial dilution, a straightforward approach is used to generate such combinations, using a two-dimensional microfluidic network as shown in Figure 1b. In the first stage, each sample was evenly and symmetrically divided into subchannels and flowed through mixing channels. The same procedure was performed in the second and third stages. The microfluidic circuit was symmetrically designed with appropriate channel lengths that could control flow rates for desired volumetric mixing ratios, to which simple electrical-circuit analysis could be applied (Figure 2). In addition, as shown in Figure 3, the resulting combinatorial mixtures could be collected by two liquid-handling methods: a pipette-based system (bottom–up) and a spotting/dropping system (top–down).

Figure 2a shows an equivalent electrical circuit for the microfluidic dilution device. In this model of the microfluidic network, an electric-fluidic analogy was used to define the proper flow rates (*Q*), pressures (*P*), and channel resistances (*R*_F_) [30]. The proposed combinatorial device is composed of six symmetrically designed microfluidic networks. As shown in Figure 2a, the highlighted channels are only calculated based on the other channel resistances given by user-defined constants. To determine the resistances of highlighted channels, we first define pressures in the microfluidic network as Equation (1) by Kirchoff’s and Ohm’s law. In this study, we used electric-hydraulic analogy based on the Hagen–Poiseuille equation. Particularly, we used the assumption that the value of the channel resistance is proportional to the length of the channel if the cross-section of the channel is identical [34,35]. Thus, the value of the channel resistance can be expressed as a relative ratio of length (or the length).

(1) $$\left(\begin{array}{l} P_{1}=R_{{\mathrm{AB}\text{-}\mathrm{AC}}_{1}}Q_{{\mathrm{AB}\text{-}\mathrm{AC}}_{1}}+R_{{\mathrm{AB}}_{3}}Q_{{\mathrm{AB}}_{3}}\\ P_{2}=R_{{\mathrm{AB}\text{-}\mathrm{AC}}_{1}}Q_{{\mathrm{AB}\text{-}\mathrm{AC}}_{1}}+R_{{\mathrm{AB}}_{3}}Q_{{\mathrm{AB}}_{3}}+R_{{\mathrm{AB}}_{4}}Q_{{\mathrm{AB}}_{4}}\\ P_{3}=R_{{\mathrm{AB}\text{-}\mathrm{AC}}_{1}}Q_{{\mathrm{AB}\text{-}\mathrm{AC}}_{1}}+R_{{\mathrm{AB}}_{3}}Q_{{\mathrm{AB}}_{3}}+R_{{\mathrm{AB}}_{4}}Q_{{\mathrm{AB}}_{4}}-R_{{\mathrm{AB}}_{6}}Q_{{\mathrm{AB}}_{6}}\\ P_{4}=R_{{\mathrm{AB}\text{-}\mathrm{AC}}_{1}}Q_{{\mathrm{AB}\text{-}\mathrm{AC}}_{1}}+R_{{\mathrm{AB}}_{3}}Q_{{\mathrm{AB}}_{3}}+R_{{\mathrm{AB}}_{4}}Q_{{\mathrm{AB}}_{4}}+R_{{\mathrm{AB}}_{7}}Q_{{\mathrm{AB}}_{7}}+R_{A_{5}}Q_{A_{5}} \end{array}\right)\;$$

(2) $$\left(\begin{array}{c} R_{{\mathrm{AB}\text{-}\mathrm{AC}}_{1}}=C_{1},\;R_{{\mathrm{AB}}_{3}}=C_{2},\;R_{{\mathrm{AB}}_{4}}=C_{3},\;R_{{\mathrm{AB}}_{6}}=C_{4}\\ R_{A_{2}}=C_{5},\;R_{{\mathrm{AB}}_{7}}=C_{6},\;R_{A_{5}}=C_{7} \end{array}\right)\;$$

In this design, (*C*_1_–*C*_7_) were set to 20 mm (*C*_1_: a micromixer in the third stage), 11.9 mm (*C*_2_: a microchannel diverged from a micromixer in the second stage), 6.1 mm (*C*_3_: a microchannel diverged from mixer in the first stage), 9.2 mm (*C*_4_: a microchannel diverged from mixer in the first stage), 9.2 mm (*C*_5_: a microchannel diverged from mixer in the first stage), 70 mm (*C*_6_: a micromixer in the first stage), 21.3 mm (*C*_7_: a microchannel for unmixed solution), respectively. In order to use the higher flow rates of output ports, groove structures were added on the serpentine mixers, which can enhance mixing efficiency in higher flow rates (Figure 2b). Thus, $R_{{\mathrm{AB}\text{-}\mathrm{AC}}_{1}}$ and $R_{{\mathrm{AB}}_{7}}$ were rewritten as Equation (3):

(3) $$\left(\begin{array}{l} R_{{\mathrm{AB}\text{-}\mathrm{AC}}_{1}}=C_{1}+\Delta R_{G}{}_{{}_{1}}\\ R_{{\mathrm{AB}}_{7}}=C_{6}+\Delta R_{G_{2}} \end{array}\right)\;$$

where $\Delta R_{G}{}_{{}_{1}}$ and $\Delta R_{G}{}_{{}_{2}}$ are the resistance values of the groove structures extruded on the serpentine mixer. Based on Equations (1) and (2), the resistances for output ports 1, 2, 4, and 5 can be calculated using Equation (4):

(4) $$\left(\begin{array}{c} R_{O_{1}}=R_{A_{1}}=\frac{P_{2}-P_{0}}{Q_{A_{1}}}=\frac{R_{{\mathrm{AB}\text{-}\mathrm{AC}}_{1}}Q_{{\mathrm{AB}\text{-}\mathrm{AC}}_{1}}+R_{{\mathrm{AB}}_{3}}Q_{{\mathrm{AB}}_{3}}+R_{{\mathrm{AB}}_{4}}Q_{{\mathrm{AB}}_{4}}}{Q_{A_{1}}}\\ R_{O_{2}}=R_{{A\text{-}\mathrm{AB}}_{1}}=\frac{P_{3}-P_{0}}{Q_{{A\text{-}\mathrm{AB}}_{1}}}=\frac{R_{{\mathrm{AB}\text{-}\mathrm{AC}}_{1}}Q_{{\mathrm{AB}\text{-}\mathrm{AC}}_{1}}+R_{{\mathrm{AB}}_{3}}Q_{{\mathrm{AB}}_{3}}+R_{{\mathrm{AB}}_{4}}Q_{{\mathrm{AB}}_{4}}-R_{{\mathrm{AB}}_{6}}Q_{{\mathrm{AB}}_{6}}}{Q_{{A\text{-}\mathrm{AB}}_{1}}}\\ R_{O_{4}}=R_{{\mathrm{AB}}_{1}}=\frac{P_{1}-P_{0}}{Q_{{\mathrm{AB}}_{1}}}=\frac{R_{{\mathrm{AB}\text{-}\mathrm{AC}}_{1}}Q_{{\mathrm{AB}\text{-}\mathrm{AC}}_{1}}+R_{{\mathrm{AB}}_{3}}Q_{{\mathrm{AB}}_{3}}}{Q_{{\mathrm{AB}}_{1}}}\\ R_{O_{5}}=R_{{\mathrm{AB}\text{-}\mathrm{AC}}_{1}}=C_{1}+\Delta R_{G_{1}} \end{array}\right)$$

If the flow rates for all output ports are identical to $Q_{\mathrm{Output}}$ ($Q_{O_{1}}=Q_{O_{2}}=Q_{O_{4}}=Q_{O_{5}}=Q_{\mathrm{Output}}$) and $P_{0}$ is zero, Equation (4) can be rewritten as Equation (5):

(5) $$\left(\begin{array}{l} R_{O_{1}}=R_{A_{1}}=\frac{R_{{\mathrm{AB}}_{3}}Q_{{\mathrm{AB}}_{3}}+R_{{\mathrm{AB}}_{4}}Q_{{\mathrm{AB}}_{4}}}{Q_{\mathrm{Output}}}+C_{1}+\Delta R_{G_{1}}\\ R_{O_{2}}=R_{{A\text{-}\mathrm{AB}}_{1}}=\frac{R_{{\mathrm{AB}}_{3}}Q_{{\mathrm{AB}}_{3}}+R_{{\mathrm{AB}}_{4}}Q_{{\mathrm{AB}}_{4}}-R_{{\mathrm{AB}}_{6}}Q_{{\mathrm{AB}}_{6}}}{Q_{\mathrm{Output}}}+C_{1}+\Delta R_{G_{1}}\\ R_{O_{4}}=R_{{\mathrm{AB}}_{1}}=\frac{R_{{\mathrm{AB}}_{3}}Q_{{\mathrm{AB}}_{3}}}{Q_{\mathrm{Output}}}+C_{1}+\Delta R_{G_{1}}\\ R_{O_{5}}=R_{{\mathrm{AB}\text{-}\mathrm{AC}}_{1}}=C_{1}+\Delta R_{G}{}_{{}_{1}} \end{array}\right)\;$$

In Equation (5), when the flow rates for output ports are fixed at the same value, the $\Delta R_{G_{1}}$ is repeated in all output ports. This results in a straightforward design rule (geometrically adding the same pattern on the serpentine channels for output ports) that does not require complex calculations for the groove structures.

In the same manner, the resistance of $R_{A_{4}}$ can be determined as Equation (6):

(6) $$\;\{\begin{array}{c} R_{{\mathrm{AB}}_{7}}=\mathrm{constant}\\ \left(\begin{array}{c} R_{{\mathrm{AB}}_{7}}=C_{6}+\Delta R_{G_{2}}\\ R_{A_{4}}=\frac{P_{4}-P_{2}}{Q_{A_{4}}}=\frac{Q_{{\mathrm{AB}}_{7}}R_{{\mathrm{AB}}_{7}}+Q_{A_{5}}R_{A_{5}}}{Q_{A_{4}}}=\frac{Q_{{\mathrm{AB}}_{7}}}{Q_{A_{4}}}C_{6}+\frac{Q_{{\mathrm{AB}}_{7}}}{Q_{A_{4}}}\Delta R_{G_{2}}+\frac{Q_{A_{5}}R_{A_{5}}}{Q_{A_{4}}} \end{array}\right) \end{array}\;$$

For easy calculation of $R_{A_{4}}$, the term of $\frac{Q_{{\mathrm{AB}}_{7}}}{Q_{A_{4}}}\Delta R_{G_{2}}$ in Equation (6) must be set to a positive integer value of a groove set. For example, $\Delta R_{G_{2}}$ is configured with four periodic groove structure sets, one of which is composed of 5 × 5 grooves. In addition, since the ratio of $Q_{{\mathrm{AB}}_{7}}$ and $Q_{A_{4}}$ is 1.5 in this design, $\frac{Q_{{\mathrm{AB}}_{7}}}{Q_{A_{4}}}\Delta R_{G_{2}}$ can be defined as 1.5 × 4 groove sets, resulting in six groove sets. 

## 3. Experimental

### 3.1. Microfabrication and Fluorescence Measurement

As shown in Figure 3, two microfluidic combinatorial devices for “top–down” and “bottom–up” with and without the groove structures, were fabricated by the multilevel SU-8 fabrication method and PDMS replica molding [36]. A master mold with two levels of SU-8 microstructures was used to make SU-8 2015 and SU-8 2050 (Microchem, Newton, MA, USA) photoresists. For the first level of 100 µm film, SU-8 2050 was spin-coated onto a silicon wafer that was selectively exposed to define the main channel. After a postexposure bake (PEB), SU-8 2015 with a thickness of 25 µm was coated onto the first level of 100 µm and exposed to UV light to pattern the groove structures. The SU-8 layer was then simultaneously developed. Another mold for the drop guide layer with 100 µm thickness was fabricated in the same manner. PDMS (Sylgard 184, Dow Corning, Midland, MI, USA) was poured onto the two molds for multilevel and drop-guide structures. After curing, the PDMS replicas were peeled off from the silicon substrates. Then, inlet and outlet holes were punched through the patterned PDMS slab. Following air-plasma treatment, the two PDMS slabs were bonded together.

For quantitative evaluation, aqueous fluorescein sodium salt (100 µg/mL in water Sigma Aldrich, Darmstadt, Germany) and distilled water were used as the sample and buffer liquids, respectively. The solutions were injected by syringe pumps with calculated input flow rates: {*Q*_A_, *Q*_B_, *Q*_C_}. A high-resolution monochrome digital camera (Hamamatsu ORCAER, Hamamatsu Photonics K.K., Shizuoka, Japan) mounted to an Olympus MVX10 epifluorescence microscope (Olympus Corporation, Tokyo, Japan) was used to capture fluorescence images, and all quantitative measurements of the fluorescent intensity were obtained using an Olympus Wasabi imaging software package (Olympus Corporation, Tokyo, Japan).

### 3.2. Computational Modeling of Concentration Profiles

The performance of the proposed microfluidic combinatorial devices was simulated by computational modeling (ESI-CFDRC, ESI Group, Paris, France). Fluids in microchannels were adopted as incompressible, laminar, miscible liquid flows with uniform properties over the computational domain. The simulation model used the steady three-dimensional conservation equations of mass, momentum, and species concentration with mathematical expressions [37].

## 4. Results and Discussion

### 4.1. Computational Fluid Dynamic (CFD) Simulation Results

**Figure 4:**
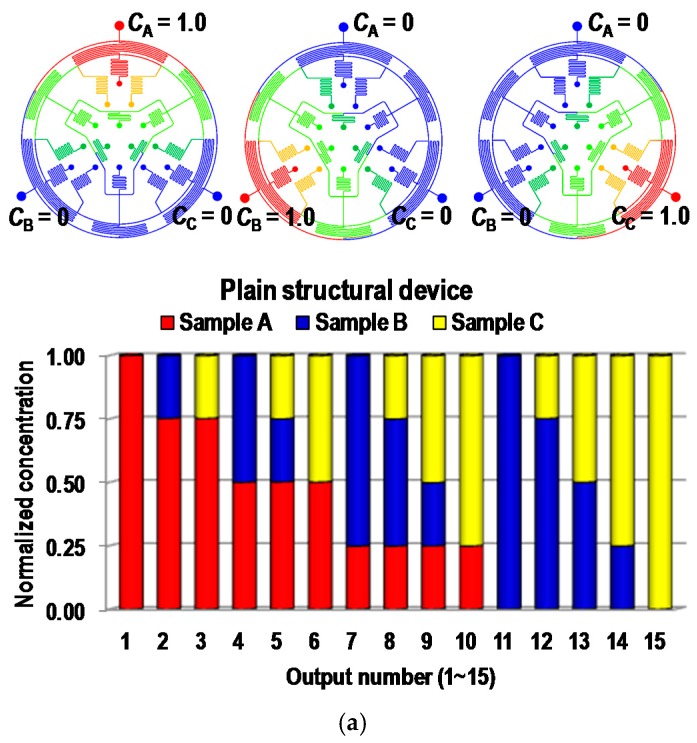

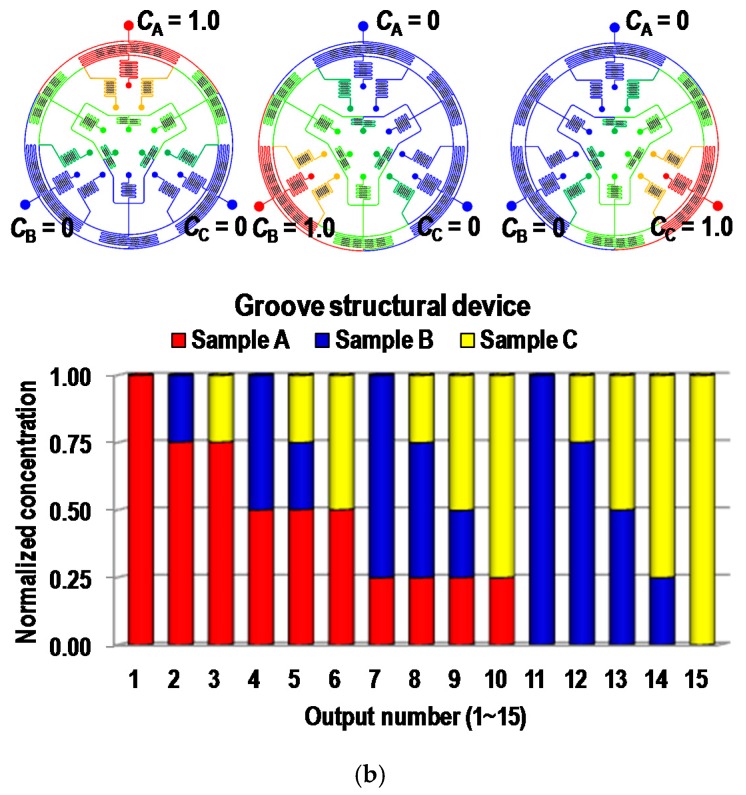
Computational Fluid Dynamic (CFD) simulation for the microfluidic combinatorial device. (**a**) Simulation result for the plain combinatorial device and (**b**) the groove structural combinatorial device. Whole simulation results were matched to the desirable values in each output combination with in 1% absolute error.

We used computational simulations to investigate the performance of the proposed microfluidic combinatorial devices. Two types of microfluidic combinatorial devices, including a plain-structural combinatorial device and a groove-structural combinatorial device were evaluated by computational fluid-dynamics software (CFD-ACE^+^, ESI Group, Paris, France) for microfluidic circuit analysis. Figure 4 shows the results of CFD-ACE simulations for the combinatorial devices without groove structures (Figure 4a) and with groove structures (Figure 4b). First, we investigated the effect of mixing efficiency enhanced by adding the groove structures. Results showed that the maximum flow rate of each sample in the plain and groove structural devices was 10 µL/min and more than 100 µL/min, respectively, assuming the diffusion coefficient of *D*_0_ is 4.4 × 10^−10^ m^2^/s in fluorescein sodium salt at room temperature [38]. Based on the conditions, two combinatorial devices were tested to investigate the combinatorial mixing performances. Only one of the three sample solutions was set to a concentration of 1.0, and the others were set to a concentration of 0. The simulation was alternatively performed with different inputs for samples A, B, and C. In all simulation cases, the input flow rates of the sample solutions were *Q*_A_ = *Q*_B_ = *Q*_C_ = 10 μL/min for the plain structural device and *Q*_A_ = *Q*_B_ = *Q*_C_ = 100 μL/min for the groove structural device. In this study, the simulations were performed under the condition of the fluids with same physical properties except for the concentration. In addition, it was based on volumetric mixing, so if the fluids were mixed properly, the concentration of each sample should have been separately close to expected values. For example, the combination of AB in Port 4 should have had a value of 0.5 when sample A was only simulated and B should also have been measured to be 0.5 in the sample condition. The results of this simulation were in good agreement with the expected values. All combinatorial devices have excellent matches with the desirable values from the mathematical modeling within 1% error for all combinations.

### 4.2. Fluorescent Experimental Results

**Figure 5:**
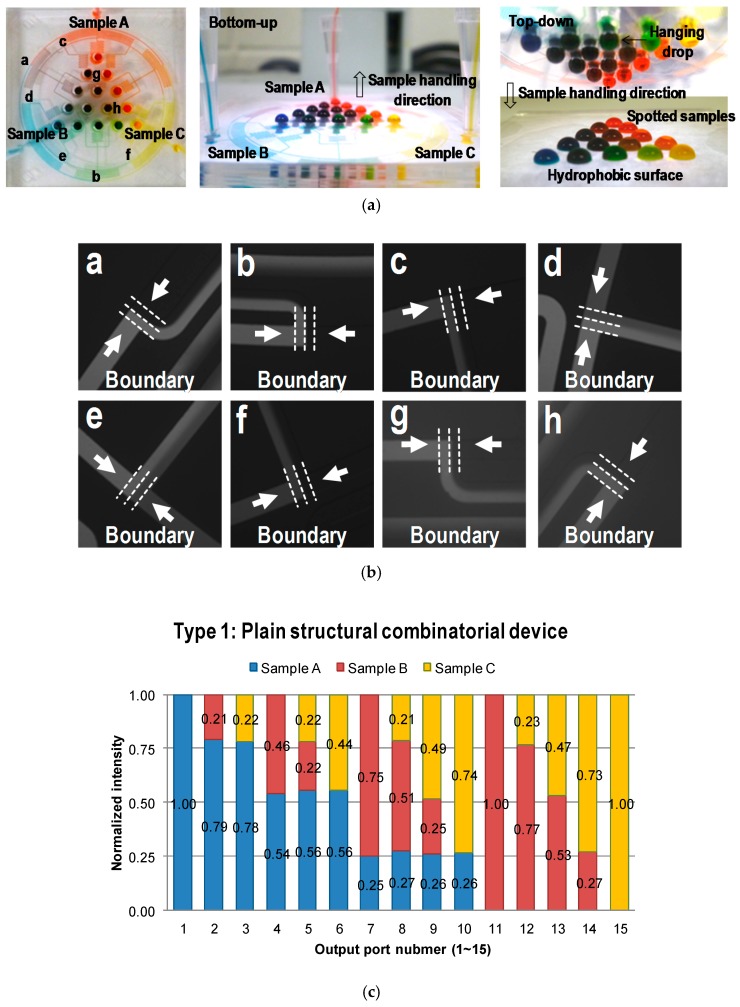

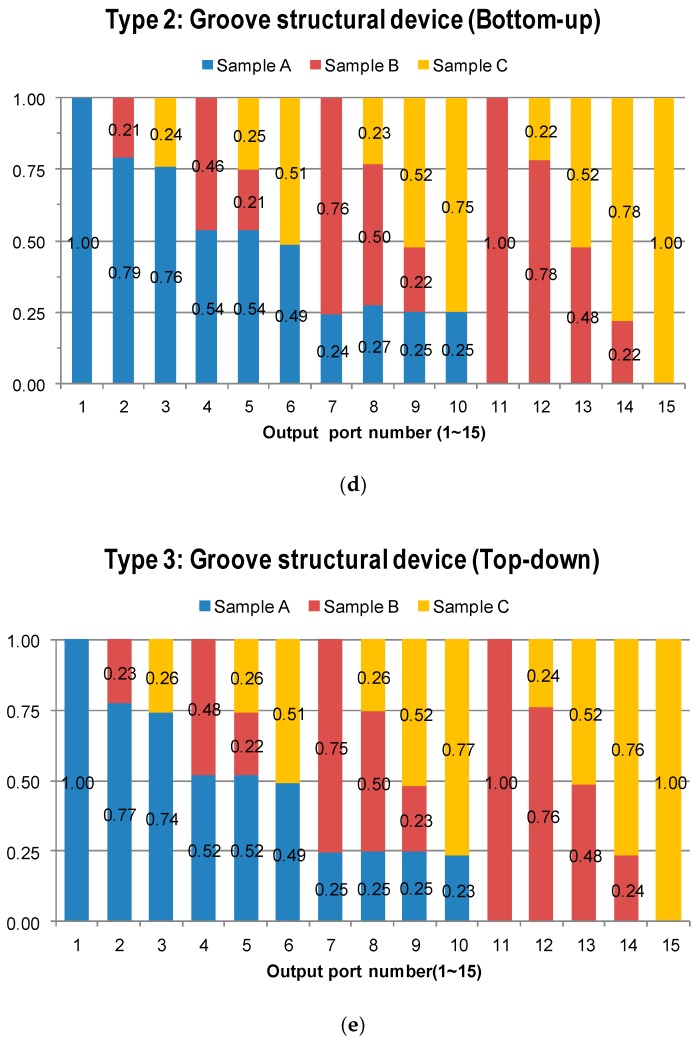
Fluorescent experimental results for the microfluidic combinatorial devices. (**a**) The images of the fabricated polydimethylsiloxane (PDMS) devices for bottom–up and top–down liquid retrievals. (**b**) Images in the mixing stages indicated in Figure 5a, the two fluids were evenly merged in the mixing stages. Results of quantitative analysis for (**c**) the plain structural combinatorial device (Type I), (**d**) the groove structural combinatorial device using bottom–up liquid-handling method (Type II), and (**e**) the groove structural combinatorial device using top–down liquid-handing method (Type III).

Two PDMS devices (plain and groove structural devices) were fabricated by soft lithography based on the simulated design, and their performances were evaluated by fluorescent analysis (sodium salt). First, the mixing performances of the two devices were tested to investigate the effect of groove structures. As a result, the maximum flow rate of Type I (plain structural device) was 10 μL/min; however, the maximum flow rate of Type II (groove structural device, bottom–up) was 100 μL/min [39,40]. Adding the groove structures resulted in enhancement of the mixing performance by more than 10 times, as discussed in the CFD simulation results. After optimization of the flow rates, only one sample was injected with the fluorescent dye (10 μL/min for Type I and 100 μL/min for Type II), and the other samples were injected with DI water at the same flow rates. Experiments were alternatively repeated under the same condition for *C*_A_, *C*_B_, and *C*_C,_ as shown in Figure 5. First, in order to arrive at a qualitative evaluation of device performance, fluidic behaviors in the mixing regions were observed. Results showed that the fluorescent solution and DI water were evenly merged as a ratio of one-to-one in the mixing regions (Figure 5b). After collecting the solutions of 15 combinatorial mixtures from the two devices, fluorescent intensities were analyzed. Supplementary Video S1 was provided to show the working principle of the proposed devices. The results for both Type I and Type II matched the desirable values within the maximum absolute error of 4%, indicating that systematically and locally arranged groove structures on mixing channels could be adapted to design the microfluidic network for the proposed combinatorial deices. We also showed another top–down liquid-handling method for spotting the resulted solution from the combinatorial devices (Type III). Type III was operated with the same conditions in a bottom–up liquid-handling method. First, samples A, B, and C were injected, and then mixed through microfluidic networks for 15 combinations. The resulted solutions were dangled on the drop-guide structure. Once the drop had been fully developed, the combinatorial device was removed from the PDMS collection substrates. Three sets of 15 mixtures from the device were sequentially collected and analyzed by the fluorescent measurement system. Figure 5e shows the combinatorial mixing results from the device. The results also corresponded to the desirable values within the 4% error. In this case, the flow rate of the input solutions was maintained at 100 μL/min per sample. When a lower flow rate was used in the operation of the device, droplets were prone to form in nonuniform sizes, and it took a long time for them to be fully developed (When the device is operated under low flow rate, droplets tend to take longer to form, and the formed droplet sizes are not uniformed). This is due to the external effects of pressure variance caused by hanging droplets on the drop guide structure. On the other hand, a high flow rate is less affected by the external-pressure variances, resulting in good performance compared to those of the lower flow rate. In addition, when the device is separated from the PDMS collection substrate, pressure variance is a critical factor for spotting systems, which is alleviated by a high level of flow rate.

## 5. Conclusions

The microfluidic network-based combinatorial dilution, covering 15 combinations with three samples for the combinatorial mixture DOE, was successfully tested by mathematical modeling, simulation, and fluidic experiments. We have also successfully demonstrated two liquid-handing methods (bottom–up and top–down). For efficient liquid-handling of the bottom–up method, flow rates in output ports were enhanced 10 times more than those of the plain type of combinatorial device (Type I) by adding the groove structure on the mixing channel (Type II). In addition, the top–down method (Type III) was also successfully demonstrated by adding the groove structure. Thus, we expect that the proposed device will be valuable in many areas of biological and material research for high-throughput screening and optimization.

## Supplementary Materials

The following are available online at http://www.mdpi.com/2072-666X/9/10/489/s1, Video S1: Working movie.mp4.
